# Editorial: Multisensory Human-Food Interaction

**DOI:** 10.3389/fpsyg.2018.00796

**Published:** 2018-05-23

**Authors:** Carlos Velasco, Kasun Karunanayaka, Anton Nijholt

**Affiliations:** ^1^Department of Marketing, Center for Multisensory Marketing, BI Norwegian Business School, Oslo, Norway; ^2^Imagineering Institute, Iskandar Puteri, Malaysia; ^3^Faculty EEMCS, University of Twente, Enschede, Netherlands

**Keywords:** food, multisensory, technology, human-food interaction, human-computer interaction, experience

In recent years, there has been a growing interest in the context of Human-Food Interaction (HFI) to capitalize on multisensory interactions in order to create, modify, and enhance our food-related experiences (Nijholt et al., 2016; Velasco et al., 2017). There are two ideas that may explain this interest. First, research has provided a strong case for the fact that eating and drinking are among the most multisensory events of our everyday lives (see Prescott, 2015; Spence, 2015, 2017, for reviews). This has paved the way to target both intrinsic and extrinsic sensory cues to design specific food experiences (e.g., Koizumi et al., 2011; Narumi et al., 2011; Spence et al., 2017). Second, given the ubiquitous nature of technology and the increasing availability of multisensory-oriented devices (Obrist et al., 2016, 2017), both researchers and practitioners have become interested in the roles that these technologies can play in food contexts (Choi et al., 2014; Petit et al., 2015).

The aforementioned ideas are the basis of the present emerging research topic, that is, Multisensory Human-Food Interaction (MHFI). Broadly speaking, research in MHFI aims to further our understanding of the principles that govern the systematic connections that exist between the senses in the context of HFI. Moreover, it also aims to build on such understanding in order to utilize and develop different technologies to “hack” the senses, that is, to modify existing, and create novel experiences, in the context of HFI (e.g., in support of healthy eating, entertainment, sensory marketing). In the present research topic, we called for investigations on these aims.

## Theme 1: multisensory principles in MHFI

Based on the idea that people associate basic tastes with music in specific ways, Wang et al. conducted a study designed to assess whether “sour music” would elicit a distinctive physiological response. In particular, their results provided evidence for the idea that salivation was greater when participants were shown an image representing a sour taste (a lemon) than when they were presented with a “sour” soundtrack or just a silent, comparable, interval of time. Note that no difference in salivation levels was found between the sour soundtrack condition compared to the silent condition. It appears that, although there might be strong links between music and tastes, music by itself is not sufficient to evoke physiological responses associated with tastes.

Reinoso Carvalho et al. investigated the effect of a beer's color on people's sensory and hedonic expectations and experience of the beer. In particular, they assessed whether dark or pale beer coloring, while keeping the taste/flavor equal, would influence people's evaluation of the beer. No differences were observed in the evaluation of the beer after tasting it, however, their participants expected to like the pale beer more than the dark one, and the latter to be more bitter, to taste stronger, and to have more “body” than the first. Here, color appears to make a difference only at the expectation level.

Taking advantage of two popular drinks, namely coffee and tea, Pramudya and Seo studied the role of temperature on people's sensory and emotional responses to such drinks. In terms of the first, their results indicated that, warmer drinks (65°C), independently of arousal, resulted in more frequent positive emotional responses. Colder drinks (5°C), on the other hand, resulted in higher activation/arousal and negative emotional responses and medium temperature drinks (25°C) resulted in more frequent low activation and negative emotional responses. As for sensory attributes, specific flavors were differentially associated with the drinks at different temperatures. For example, “roasted flavor” attribute of brewed coffee was more often associated with the high temperature condition (65°C), while characteristics such as “pungent aroma,” “metallic flavor,” and “skunky flavor” were more often reported in relation to the lower temperature (5°C). Therefore, temperature can influence both sensory and emotional responses to drinks.

Fenko et al. also used coffee as a means to assess the effect of extrinsic cues on flavor and more broadly on product evaluation. In particular, they assessed the influence of visual metaphors (a lion located on top or bottom of a coffee packaging) and textual claims of strength, on the evaluation of coffee products. Their results demonstrated that both elements can influence coffee expectations and experience. For example, both the textual claim (“extra strong”) and visual metaphor (image located on the bottom) of strength enhanced the perception of coffee strength and also the participants' intention to purchase the product. Therefore, visual characteristics of a drink's packaging but also any textual information used to describe it, may influence its expectations and experiences.

Through a metanalytical approach, Roque et al. presented a comprehensive review on the concept of freshness in beverages. Using multisensory flavor perception as a frame of reference, the authors suggested that freshness perception is characterized by both perceptual and semantic elements. They differentiated it from flavor by indicating that freshness is more specific in terms of the particular functions that it serves, such as to alleviate oropharyngeal symptoms. Moreover, they also suggested that the weighting of different sensory inputs in freshness also differs from that of flavor.

Broadening the understanding of multisensory principles and the use of new technologies in MHFI, Zhao et al. conducted a study to assess people's visual search behavior for wine bottles with characteristic triangles in their label. Both of the experiments in their study were conducted in virtual reality. Consistent with previous literature, their results revealed that participants identified faster bottles with downward vs. upward pointing triangles, and that this effect was modulated by the bottle's location on the shelf. Their results provided relevant information as to how extrinsic attributes (e.g., label elements) influence wine search. Moreover, Zhao et al. forwarded virtual reality means to assess product performance.

## Theme 2: developing and utilizing multisensory technologies in HFI

Aoyama et al. presented a study designed to further understand the way in which galvanic tongue stimulation influences tastes sensations. First, they tested the effect of taste inhibition for cathodal GTS in non-electrolytes aqueous and electrolytes aqueous solutions.  Ayoma et al. found that cathodal GTS (1.0 mA and 2,000 ms square current) only weakened the sweet and bitter taste sensations in electrolytes aqueous solutions (which are glycine and MgCl_2_). In the second experiment, they investigated whether the cathodal-GTS would inhibit all five basic tastes. Their results showed that all tastes sensations were weakened by cathodal-GTS. Further, this effect was strongly correlated with the strength of the current. The findings of this study support the ion migration hypothesis for taste inhibition as cathodal-GTS only weakened the sensations produced by electrolytes aqueous solutions. This paper informs research on digital flavor technologies.

Risso et al. developed and tested a practical small-sized computer-controlled olfactometer, or Multi-Fragrance Olfactory Display (MFOD), that can be used in food and beverage research. This olfactometer used a solid fragrance release method to produce smell sensations and it had eight different odor channels. The intensity and flow rate of the fragrances were adjusted by changing the speed of the centrifugal fan and using a small anemometer. The results of their study confirmed that this olfactometer can significantly modulate the participants' evaluation of foods and beverages. This miniaturized portable olfactometer provide a low cost and efficient odor delivery solution for food and beverage experiments.

Finally, Velasco et al. contributed with a mini-review on multisensory technologies that have been forwarded both for flavor, but also more general, food and drink augmentation. Whilst their suggestion was that there are a number of interesting of these technologies that promise to transform HFI design (e.g., experience design, healthy eating, and sensory marketing), they also indicated that there are several challenges that will need to be addressed carefully before they become part of our everyday life food and drink experiences.

Overall, the research presented in this topic contributes to both multisensory principles and technologies in MHFI. We hope that the papers featured in this special issue will support and inspire further research in MHFI.

## Author contributions

All authors listed have made a substantial, direct and intellectual contribution to the work, and approved it for publication.

### Conflict of interest statement

The authors declare that the research was conducted in the absence of any commercial or financial relationships that could be construed as a potential conflict of interest.
